# Percutaneous ureteroscopy laser unroofing-a minimally invasive approach for renal cyst treatment

**DOI:** 10.1038/s41598-017-14605-4

**Published:** 2017-10-31

**Authors:** Jia Hu, Najib Isse Dirie, Jun Yang, Ding Xia, Yuchao Lu, Xiao Yu, Shaogang Wang

**Affiliations:** Department of Urology, Tongji Hospital, Tongji Medical College, Huazhong University of Science and Technology, Wuhan, 430030 China

## Abstract

Most simple renal cysts rarely require therapy. When it grows to such a large size, regardless of the presence of symptomatology, surgical intervention is required. In this study, we explored a new approach called percutaneous ureteroscopy laser unroofing for treatment renal cysts and evaluated its safety and efficacy. 71 simple renal cyst patients with surgical indications were enrolled, including 6 patients with a peripelvic cyst and 5 patients coexisting ipsilateral calculi. Under ultrasound guidance, an eighteen-gauge needle was placed inside the cyst cavity, and a guidewire was introduced followed by sequential dilation up to 28 F. The extra-parenchymal portion of cyst wall was dissociated and incised using either a Thulium or Holmium laser, and a pathological examination was performed. Renal calculi were treated simultaneously. For peripelvic cyst patients, one end of a double-J stent was inserted into the cyst cavity to prevent auto-closure. Mean of 11.7 months follow-up, the results showed that the cyst was completely resolved in 53 patients, its size was reduced to less than 50% in 15 patients, and treatment failed in only 3 anterior cyst patients, suggesting that percutaneous ureteroscopy laser unroofing is an effective and less invasive alternative for treatment of renal cysts in selected patients.

## Introduction

Renal cysts are simple benign lesions with an unknown etiology that are either inherited or acquired. The kidney is one of the most common sites of cyst formation in the human body; the estimated prevalence of renal cysts is approximately 10% of the general population, and the incidence increases with age^1,2^. Most renal cysts are simple cysts and are asymptomatic, and many of them are found incidentally through routine physical examinations and rarely require therapy^3^. However, when the diameter of the cyst is >5 cm and renal parenchyma (collection system) compression is present, regardless of the presence of flank or abdominal pain, surgical intervention is required^4^.

A variety of therapeutic interventions have been described for benign symptomatic cystic lesions of the kidney. These include cyst aspiration^5^, surgical resection^6,7^, and sclerotherapy with a variety of different agents^8,9^. Although none of these approaches appear to be better than the others described, aspiration and sclerotherapy are associated with a higher incidence of cyst recurrence, fever, and ureteropelvic junction obstruction regarding the treatment of peripelvic cysts, given their proximity to the renal vessels and collecting system. These techniques may also require multiple treatments to satisfactorily ablate the cystic lesion^5,10^.

A laparoscopic approach for renal cyst unroofing was the first described in 1992 and has since become the gold standard of treatment. Laparoscopy may be associated with a higher safety margin and better efficacy, especially for peripelvic cyst treatment^11,12^. However, this approach requires general anesthesia, which causes an increase in hospitalization expenses and an extension of the postoperative hospital stay. In addition, three ports are needed for the procedure, which makes it controversial as an ideal minimally invasive treatment.

Recently, we have explored a new approach for the treatment of symptomatic renal cystic lesions that merges the merits of both of the abovementioned methods and is referred to as percutaneous ureteroscopy renal cyst laser unroofing. This significantly less invasive approach needs only one port, is more comfortable for patients, and requires less expensive anesthesia than a laparoscopic procedure. Moreover, this approach is more attractive if there are coexisting calculi that also require treatment. In this study, we sought to assess the efficacy and safety of this procedure in the management of patients with symptomatic renal cysts or cysts complicated with ipsilateral renal calculi.

## Results

The operations were performed successfully in all patients and their characteristics are given in Table 1. No serious complications occurred intraoperatively or postoperatively, such as active bleeding, urinary leakage or injury of the renal parenchyma and adjacent organs. Postoperative moderate or above pain according to visual analogue scale was not observed in all of patients. Some of these patients suffered mild pain and were rated as Clavien grading score 1, which required or not analgesic treatment. Other Postoperative complications included fever in 2 patients (2.8%) that were rated as Clavien grading score 2 and required antibiotic treatment.

**Table 1:** Perioperative and Follow-up Results.

Variable	Data
No. of patients	71
Mean Operative time (range) (min)	35.3 (24–68)
Mean Hospital stay (range) (d)	3.1 (1–4)
Conversion to open surgery (n)	None
Perioperatively serious complications	None
Mean Follow-up (range) (m)	11.7 (3–24)
**Radiographic evaluation**	
*Renal cysts*	
Resolved, n (%)	53 (74.7%)
>50% Reduction, n (%)	15 (21.1%)
Recurrence, n (%)	3 (4.2%)
*Stone clearance, n (%)*	5 (100%)

The mean operative time was 35.3 minutes (range, 24 to 68 minutes); the operative times were obtained from the anesthesia record and were recorded as the time from the initial ultrasound-guided puncture or cystoscopy to the placement of the ureter stent until the placement of the nephrostomy tube. The cyst fluid cytology was negative for malignancy, and the histopathologic diagnosis of all cyst walls was consistent with a simple renal cyst. Some patients with peripheral or peripelvic cysts were usually discharged 1–2 days after the surgery, while others required the pathological results before discharge, so the postoperative hospital stay would be exceeded. In addition, Patients with renal cysts complicated with ipsilateral renal calculi were hospitalized for a relatively long time, usually 2–4 days after the surgery. Therefore, the mean postoperative hospital stay was 3.1 days.

**Figure 1:**
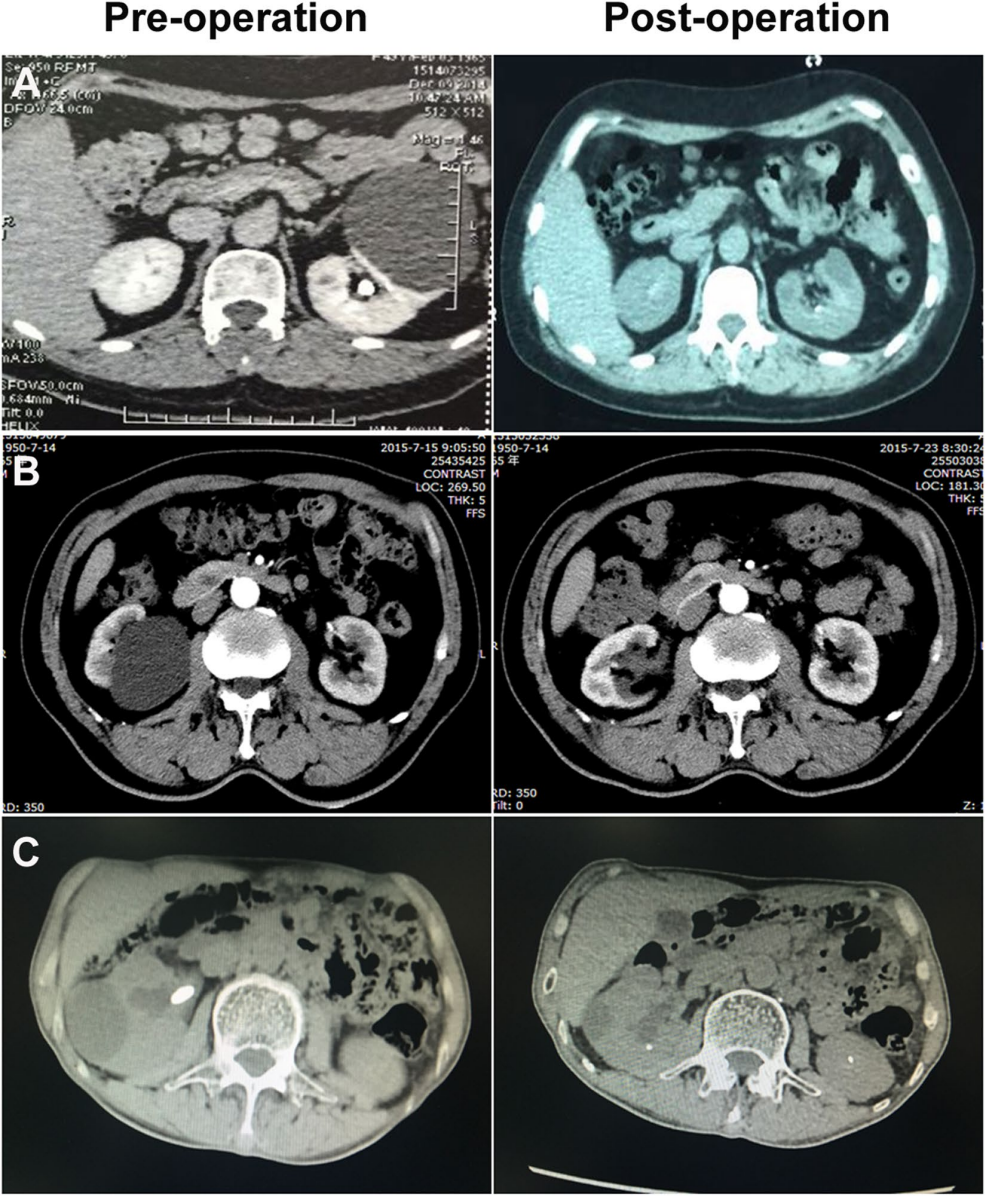
Preoperative and postoperative computed tomography (CT) for patients with peripheral cysts **(A**), patients with peripelvic cysts **(B**) and patients with renal cysts complicated with ipsilateral renal calculi **(C**).

The patients were followed up at least every 3 months for the first year and annually thereafter. With a mean follow-up of 11.7 months (range, 3–24 months), all patients underwent an abdominal CT scan or bilateral ultrasonography of the kidneys. It was slightly difficult to persuade the patient to return for a follow-up due to the benign nature of the disease. Some patients came after two years, but others managed to come after 6 or 9 months postoperatively. Of the 71 cyst units, 53 cyst units (74.7%) were completely resolved with almost 100% disappearance of the cyst, and 15 cyst units (21.1%) were reduced in size by more than 50% compared to the preoperative size (Fig. 1). However, treatment failed in 3 patients with anterior cysts (4.2%), and the cysts persisted completely after 3, 6 or 9 months of follow-up, probably due to an incomplete resection. Follow-up CT or ultrasound imaging also revealed a complete resolution of renal calculi in patients with a renal cyst complicated with ipsilateral renal calculi.

## Discussion

Renal cysts remain the most common types of benign renal lesions, and represent more than 70% of asymptomatic renal masses^2,13^. Regarding the risk factors for the development of cysts, increasing age, male gender, the presence of hypertension, and the presence of renal insufficiency were all associated with the development of sporadic renal cysts^14^. The overwhelming majority of simple or minimally complex cysts (Bosniak class I, II, and IIF cysts) are likely to represent benign lesions and require either no therapy or just continued radiographic follow-up in patients with a diagnosis of class IIF lesions^15,16^. However, benign cystic lesions of the kidney can grow to such a large size (diameter >5 cm) that they may cause flank (abdominal) pain and constrict the nephrons; in these cases, an intervention will become necessary^4,7,17^.

The use of antegrade percutaneous ureteroscopy laser unroofing to treat simple renal cysts is a novel technique that has been explored by clinicians at our center. With this approach, surgery was performed on 71 patients, and the mid-term follow-up results showed that the cyst volume shrank in 68 patients and the recurrence rate was only 4.2%. That relatively low recurrence rate is similar to the rates reported by several groups using a laparoscopic approach, which were approximately 4.5–5.6%^18,19^. The recurrence rate associated with percutaneous aspiration and sclerotherapy was up to 82%^5,20^.

One of the strengths of this technique is that it requires just a single incision. This technique was associated with a similar recurrence rate as a traditional laparoscopic approach, it was minimally invasive and showed superior cosmetic results. The established 28F work channel can accomplish most of the cyst wall dissociation, as seen with a laparoscopic approach. When the sheath and ureteroscope both returned to the exterior cyst during the operation, perirenal adipose tissue that adhered to the exterior cyst wall was identified. Then, by using a ureteroscope and a hydro-dissection technique, the extra-parenchymal portion of the cyst wall could be separated from the surrounding perirenal adipose tissue. The tensility was maintained, and the collapsed cyst wall was grasped and resected in the Amplazt sheath interior, which can ensure a safe incisal margin and avoid injury to the normal renal parenchyma. One deficiency of the technique was that once the cyst is grasped, the size of cyst wall is limited, and good effects can be achieved by repeating the grasp and resection procedure. The Thulium and Holmium laser are most commonly used lasers in our center for cyst wall resection. The Thulium laser has a higher cutting efficiency than does the Holmium laser^21^, whereas the latter possess two characteristics: cutting and lithotrity.

Another attractive advantage of this approach is the simultaneous treatment of renal cysts and ipsilateral calculi for selected patients. Our experience is that the optimal patient is one who has a solitary, posterior, large-sized renal cyst and ipsilateral calculi, and our priority is to achieve stone clearance. If the renal cyst was not on the optimal puncture route, we would choose an additional route to reach the cyst after the percutaneous nephrolithotomy (PCNL). In our study, 5 renal cyst patients with coexisting ipsilateral calculi showed a good therapeutic effect, which could decrease the costs and the risks associated with a potential second operation. This viewpoint is further supported by a recent study by Chen *et al*., who described percutaneous intrarenal marsupialization and concomitant nephrolithotomy for the treatment patients with a renal cyst and ipsilateral calculi and follow-up results have confirmed the safety and efficacy of the procedure^22^. Gelet A *et al*. first reported in 1980 that the use of antegrade percutaneous resection offers an excellent surgical alternative for renal cysts and is associated with a 9% recurrence rate according to follow-up studies^23,24^. First, a 30F work channel was established, a standard transurethral resectoscope was used to resect the exophytic portion of the cyst wall and the parenchyma portion of the cyst was subsequently cauterized. However, this approach cannot simultaneously treat patients with a renal cyst that coexists with ipsilateral calculi. In addition, it used distilled water, not saline used in our approach as an intraoperative irrigation fluid, which may increase the potential incidence of dilutional hyponatremia in patients.

It is worth noting that we are the first to demonstrate that ultrasound-guided paravertebral block (PVB) is an attractive option for anesthetic management during PCNL^25^. We also performed it to offer anesthetic cover for percutaneous ureteroscopy renal cyst laser unroofing, which could theoretically become a daytime operation. Compared to general anesthesia, PVB anesthesia can decrease both the costs and the incidence of postoperative malignant vomiting attributed to a reduced use of opioids^26^ and can retain the patient’s muscular strength in the lower limbs, which accelerates their recovery^25^.

A retrograde approach with flexible ureteroscopy laser marsupialization is another novel technique with which to manage renal cysts. Kavoussi *et al*. initially described the transurethral management of a peripelvic renal cyst^27^. The incision in the cyst wall created a permanent channel between the renal collecting system and the cyst, which forces the cyst fluid to continuously drain into the pelvis. Other colleagues have reported on the safety and efficacy of this approach^28,29^. Admittedly, the natural orifice transluminal endoscopic surgery (NOTES) is the least invasive approach. Unfortunately, this technique is limited in the treatment of peripelvic cysts that coexist with ipsilateral calculi and does not enable the collection of enough tissue specimens for histopathologic analysis.

Some limitations of our report need to be acknowledged. Patients with a solitary, larger posterior or parapelvic renal cyst that has an appropriate puncture route are ideally suited for the surgery. However, it is not recommended for patients with an anterior cyst. Due to the limited dissociation range, the extra-parenchymal portion of the cyst wall is very difficult to manipulate, which can cause incomplete resection and high recurrence rates (27.3%, 3/11), as seen during the intermediate follow-up in our study. This viewpoint is supported by studies by Hamedanchi, S *et al*.^24^ and Chen *et al*.^22^. Other limitations to the study include the single-center retrospective analysis, the relatively small sample size and the short-term follow-up period. Moreover, the prolonged placement time of the nephrostomy tube and the double-J stent in patients with a peripelvic cyst may slightly increase the degree of stent-related symptoms.

To sum up, percutaneous ureteroscopy laser unroofing offers several advantages over a laparoscopic approach for treating patients with a solitary, larger posterior or parapelvic renal cyst; this technique is minimally invasive, can simultaneously address ipsilateral calculi, possibly lower the costs and result in a shorter hospital stay. In addition, the instruments that are used are familiar to the urologist, and the technique can be performed in any endourological center. However, the few limitations of this study still merit consideration. A multi-center, randomized, prospective, control study with a large sample and a long follow-up time is needed to validate the results of this exploratory study in the future.

## Methods

### Patients

This present study was approved by the Ethical (Helsinki) Committee of Tongji Hospital of Tongji Medical College of Huazhong University of Science and Technology. All study participants provided written informed consent. All the methods performed in patients were carried out in accordance with the relevant guidelines and regulations. From November 2014 to October 2016, patients who had surgical indications and simple renal cysts were evaluated via abdominal ultrasonography and contrast-enhanced computer tomography CT. According to the Bosniak Classification system of renal cyst lesions, 71 patients with a class I and II cyst lesions were enrolled in the study. In addition, patients with calcification, septation or enhancement of their renal mass and coagulation disorders were excluded from the study. In regards to the 71 patients, 46 complained of flank and abdominal pain, and 25 patients exhibited a renal cyst by routine imaging examination. Of all 71 patients, 19 had cysts that were in the upper pole, 23 had cysts that were in the middle pole, 22 had cysts in the lower pole and 6 patients had peripelvic cysts. The mean diameter of the cysts was 6.5 cm (4.9–9.6 cm) and 5 patients had a renal cyst that was complicated with ipsilateral renal calculi. The mean stone surface area was 5.7 cm^2^ (3.4–9.8 cm^2^). Three patients had a history of ipsilateral renal cyst operation (2 cases received laparoscopic renal cyst unroofing and 1 case received percutaneous aspiration). The operative procedure was carefully explained to each patient, and the possible complications and the invasiveness of the procedure were detailed. Patients were also informed of and offered alternative treatment methods, including the laparoscopic unroofing procedure. Patients were actively involved in the decision-making process, and their preferred treatment option was chosen. This manuscript are not contains information or images that could lead to identification of the study participant. Characteristics of the patients are given in Supplementary Table 1.

### Anesthesia

All patients underwent continuous epidural anesthesia or ultrasound-guided paravertebral block anesthesia. The procedures for the latter were as follows:

The patient was firstly placed in a supine position. Hydromorphone 1 to 2 mg was administered intravenously 10 minutes prior to the nerve block for patient’s comfort during puncture. The patients were then turned to lateral position with the side to be operated upward. The level of nerve block was T10/T11, T11/T12, and T12/L1. Ultrasound-guided PVB was performed using a linear array US 5- to 10-MHz probe. The probe was positioned parallel to the rib through the lumbar back. With the probe being moved cephalad or caudad, the transverse process of T 10, T11, T12 or L1 was identified. After infiltration of 2% lidocaine, a 100-mm, 21-gauge Tuohy needle (SonoPlex Nanoline; Pajunk Inc, Geisingen, Germany) was respectively introduced into the T10/T11, T11/T12, and T12/L1 paravertebral spaces under direct visualization of the needle tip. 7 ml to 10 mL of 0.5% ropivacaine was injected at each segment.

### Surgical technique

**Figure 2:**
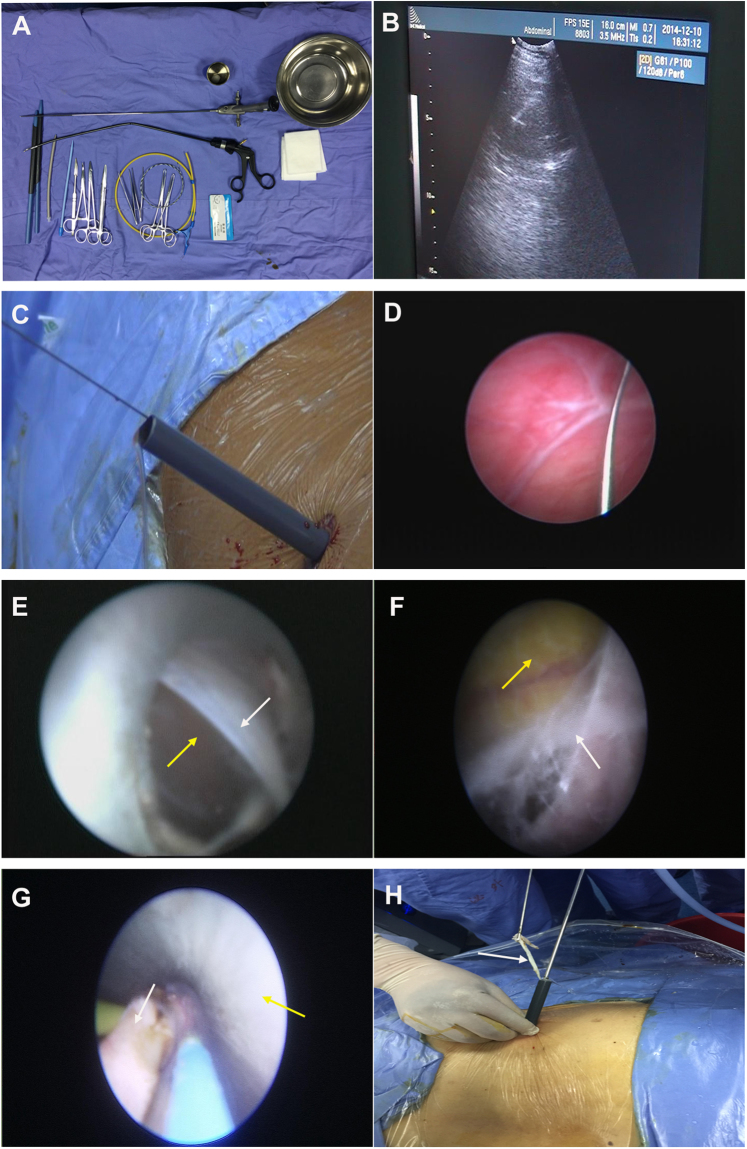
The surgical procedures for percutaneous ureteroscopy laser unroofing in the management of renal cysts. **(A)** Surgical instruments for percutaneous ureteroscopy laser renal cyst unroofing. **(B)** A needle was placed inside the cyst cavity under ultrasound guidance. **(C)** Using fascial dilators, a working channel was dilated in a sequential fashion up to 28F. **(D**) The interior cyst was inspected with an 8/9.8F rigid ureteroscope. **(E)** The sheath and the ureteroscope both returned to the exterior cyst (yellow arrow), then reached a proper plane between the extra-parenchymal portion of the cyst (white arrow) and perirenal adipose. **(F**) The exterior cyst wall (white arrow) was dissociated from perirenal adipose tissue (yellow arrow). **(G**) The cyst wall (white arrow) was grasped and pulled towards the Amplazt sheath interior (yellow arrow). **(H)** A circumferential incision of the cyst wall (white arrow) was made with a laser and was pulled out.

Surgical instruments are similar to those used in percutaneous ureteroscopy laser lithotripsy (Fig. 2A). The surgical procedures were as follows:

### Patients with peripheral cysts

Patients in this group were then placed in the prone position for percutaneous puncture and tract dilation. Under ultrasound guidance, the cyst was identified and an eighteen-gauge access needle (Cook, Bloomington, USA) was placed inside the cyst cavity (Fig. 2B). The contents of the cysts were aspirated for cytological and bacteriological analysis. A guidewire was introduced through the needle cannula and inserted into the cyst cavity, followed by tract dilation with fascial dilators in a sequential fashion up to 28F. Finally, a 28F Amplazt sheath was placed inside the cyst cavity (Fig. 2C). An 8/9.8F rigid ureteroscope (Karl Storz, Tuttlingen, Germany) connected to a normal saline irrigant was inserted through the Amplazt access sheath and advanced into the cyst cavity, and the interior of the cyst was inspected (Fig. 2D). The guidewire was retained in the interior cyst, then the sheath and ureteroscope both returned to the exterior of the cyst and reached a proper plane between the extra-parenchymal portion of the cyst wall and the surrounding perirenal adipose (Fig. 2E). The exterior cyst wall was dissociated as much as possible from perirenal adipose tissue with a ureteroscope and irrigation pump pressure (150~250 mmHg) (Fig. 2F). The actions were performed gently, avoid injury of the renal parenchyma. A portion of the collapsed cyst wall was grasped and gently pulled towards the Amplazt sheath interior with either laparoscopic or cystoscopic grasping forceps (customized curved) (Fig. 2G). A Thulium Laser (Vela® XL power 40~50 W) or a Holmium laser (Lumenis Corporation, USA power 60~70 W) was introduced through the ureteroscopic working channel, a circumferential incision of the cyst wall was then made while pulling the cyst tissue out until it was nearly completely removed (Fig. 1H). Finally, a 22 F nephrostomy tube was placed after the procedure, and the cyst wall tissue was sent for histopathologic analysis. All patients were discharged 1–3 days after the surgery, and the nephrostomy tube was removed prior to discharge. The brief surgical procedure showed in Supplementary Video 1.

### Patients with peripelvic cysts

**Figure 3:**
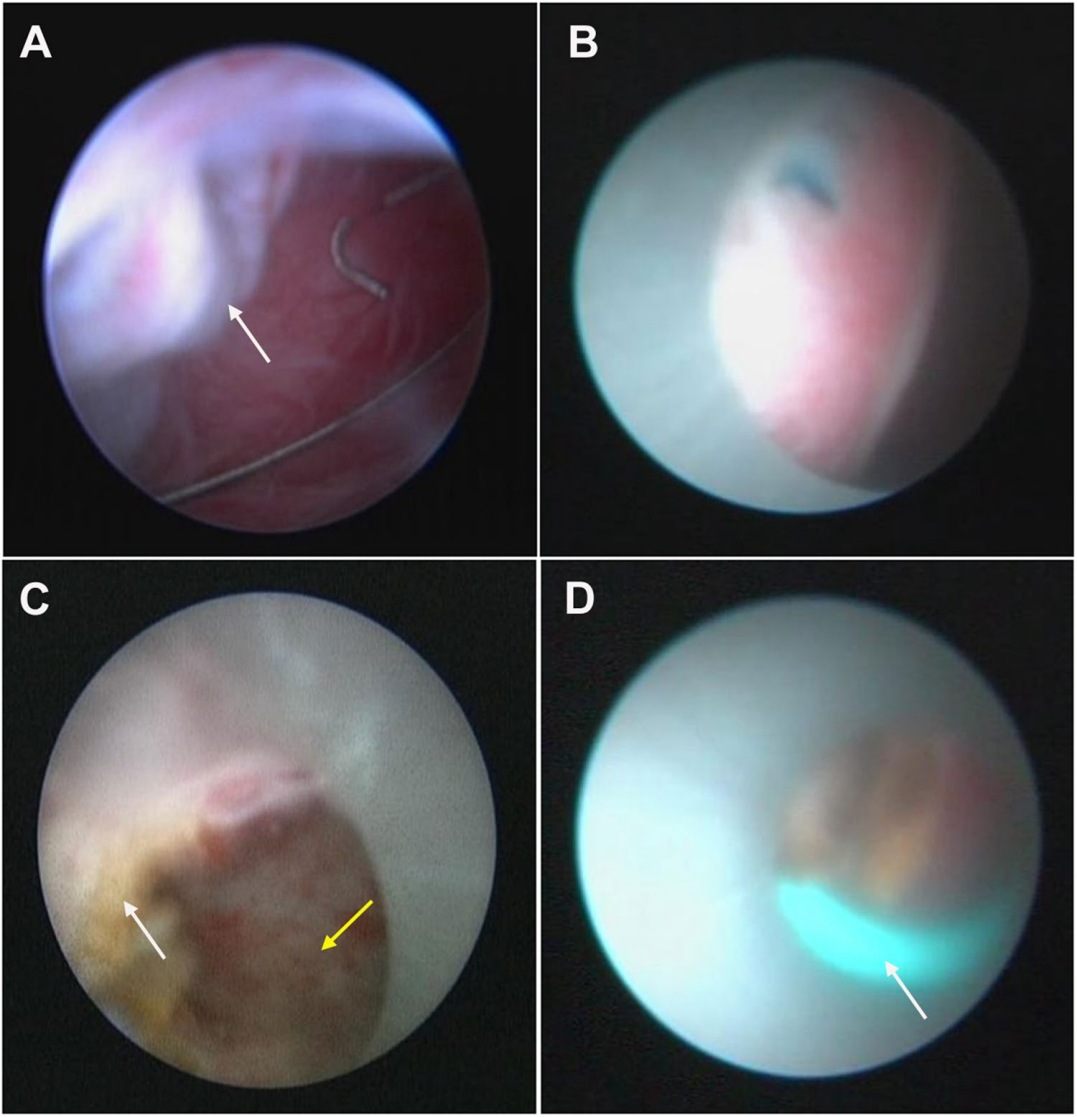
The surgical procedures for percutaneous ureteroscopy laser unroofing in the management of peripelvic cysts. **(A**) A ureteroscope was used to identify the wall (white arrow) adjacent to the renal pelvis. **(B)** The suspicious interior wall was punctured by the needle. **(C**) The wall was incised with a laser to create a permanent connection between the cyst (white arrow) and the adjacent renal collecting system (yellow arrow). **(D**) One end of a 6 F double-J stent (white arrow) was inserted into the cyst cavity.

Patients with peripelvic renal cysts were first placed in the dorsal lithotomy position, where the cystoscope was then used to identify the target ureter orifice, an open-ended 6 F ureter stent was then placed through the cystoscope and into the renal pelvis (used for blue methylene injection to aid in the subsequent identification of the renal pelvis). Patients were then placed in a prone position as in the first group. Under ultrasound guidance, the peripelvic cyst was identified, and then an eighteen-gauge needle cyst puncture was made. Tract dilation was started and was dilated up to 24F; excessive tract dilation was thought unnecessary and was avoided to prevent possible bleeds due to its closeness to a highly vascular area compared to peripheral cysts. After effective dilation, an ureteroscope was used to visualize the cyst cavity and to identify the interior wall adjacent to the renal pelvis (Fig. 3A). Then, the suspicious wall was punctured with a needle for further clarification (Fig. 3B). A Thulium or Holmium laser was used to incise a 2–3 cm wall to create a permanent communication between the cyst and the adjacent renal collecting system (Fig. 3C). At the end of the procedure, we inserted one end of a 6F double-J stent into the cyst cavity to prevent auto-closure for a maximum of two months (Fig. 3D) and left the 20F nephrostomy tube in the renal pelvis for two weeks.

### Patients with renal cysts complicated with ipsilateral renal calculi

PCNL and cyst unroofing were performed simultaneously on all of the five patients with renal cysts that coexisted with ipsilateral renal calculi. The puncture procedure and tract dilation targeting the stone through the cyst was created and dilated up to 28F. After fragmenting and extracting the stone, the same unroofing procedure described above was used to treat the cyst. Of the five patients, one required an additional puncture route to reach the cyst after the PCNL due to the far distance between the cyst and the stone. In the other patients, all of the procedures were performed with a single puncture route.

## Electronic supplementary material

Supplementary video 1

Supplementary table 1
