# Supplementary material for: Percutaneous ureteroscopy laser unroofing-a minimally invasive approach for renal cyst treatment
Source: Sci Rep. 2017 Oct 31;7:14445. doi: 10.1038/s41598-017-14605-4 (PMC5663957; doi:10.1038/s41598-017-14605-4)
Supplement: Supplementary file 2 — Supplementary table 1 [file 41598_2017_14605_MOESM2_ESM.pdf]

Percutaneous ureteroscopy laser unroofing-a minimally invasive approach for renal cyst treatment

Jia HU, Najib Isse DIRIE, Jun YANG, Ding XIA, Yuchao LU, Xiao YU* and Shaogang WANG*

Department of Urology, Tongji Hospital, Tongji Medical College, Huazhong University of Science and Technology, Wuhan 430030, China

* To whom correspondence should be addressed: Xiao YU or Shaogang WANG
Department of Urology, Tongji Hospital, Tongji Medical College, Huazhong University of Science and Technology, Liberalization Ave, No. 1095, Wuhan 430030, P.R. China.
Tel: 86-27-83663460; E-mail: yujiuhu@163.com or sgwangtjm@163.com Tel & Fax: +86-27-83663460

Supplementary table 1. Patient characteristics

Variable	Data
**No. of patients**	**71**
**Sex**	
Male,n (%)	42 (59.1%)
Female,n (%)	29 (40.9%)
**Mean age (range) (y)**	47.4 (34-71)
**Body mass index, kg/m^2**	26.1 (22.5-37.3)
**Cystside:**	
Right,n (%)	37 (52.1%)
Left, n (%)	32 (45.1%)
Bilateral, n (%)	2 (2.8%)
**No. of cysts**	
1, n (%)	63 (88.7%)
>1,n (%)	8 (11.3%)
**Cyst location**	
Upper,n (%)	19 (26.7%)
Middle,n (%)	23 (32.4%)
Lower, n (%)	22 (31.0%)
Parapelvic, n (%)	7 (9.9%)
**Cyst with ipsilateral renal calculi number**	5 (7.0%)
**Cyst position**	
Anterior, n (%)	11 (15.5%)
Posterior, n (%)	60 (84.5%)
**Clinical symptoms:**	
Flank or abdominal pain,n (%)	46 (64.8%)
Renal lump,n (%)	25 (35.2%)
**Maximum diameter of cyst, cm (range)**	6.5（4.9-9.6）
**Stone surface area, cm^2 (range)**	5.7 (3.4-9.8)
**Bosniak classification:**	
I, n (%)	64 (90.1%)
II,n (%)	7 (9.9%)
**Previous operation, n**	3 (4.2%)
